# Evaluation of Accuracy and Reproducibility of an AI-Based Cephalometric Landmark Detection Software: A Comparative Study with Manual Annotations

**DOI:** 10.4317/jced.64030

**Published:** 2026-05-29

**Authors:** Afaf El Merouani-Drissi, Hajar Ben-Mohimd, Afaf Alassfar, Douae Ahmadoun, Fatima Zaoui, Hicham Benyahia

**Affiliations:** 1 Resident, Department of Orthodontics, Faculté de Médecine Dentaire, Université Mohammed V de Rabat; 2 Assistant Professor, Department of Orthodontics, Faculté de Médecine Dentaire, Université Mohammed V de Rabat; 3 PhD, Independent Researcher, Paris; 4 Professor and Head of Department, Department of Orthodontics, Faculté de Médecine Dentaire, Université Mohammed V de Rabat; 5 Professor, Department of Orthodontics, Faculté de Médecine Dentaire, Université Mohammed V de Rabat

## Abstract

**Background:**

Automated cephalometric landmark detection using artificial intelligence (AI) has emerged as a promising aid in orthodontic diagnosis and treatment planning. However, its clinical usefulness depends on both accuracy of detection and quality of the reference annotations that are used for evaluation. Objective: To assess the accuracy, precision and stability of an AI-based cephalometric landmark detection system (WebcephTm) using a rigorously validated ground truth.

**Material and Methods:**

One hundred lateral cephalograms were independently annotated by expert orthodontists. A custom-developed application was used to extract and standardize landmark coordinates in millimeters from radiographic images. Inter-observer agreement validated the ground truth. AI-annotated landmarks were compared with expert annotations using Euclidean distance measurements. Landmark-dependent variability was examined, and the stability of AI behavior across images was evaluated using the coefficient of variation (CV).

**Results:**

Inter-observer analysis demonstrated excellent agreement (ICC=1; mean discrepancy 1,53+- 0.70 mm), confirming the reliability of the reference. The AI system showed a mean localization error of 3,16 mm, with only 42,7% of detections within the 2-mm clinical threshold. Performance was strongly landmark-dependent. Soft-tissue landmarks showed higher localization errors than skeletal landmarks, although this difference was not statistically significant (Mann-Witney U test, p &gt; 0,05). CV analysis confirmed that AI errors were anatomically driven rather than randomly distributed.

**Conclusions:**

AI-based cephalometric detection by WebcephTm is reliable for well-defined skeletal landmarks, while soft-tissue points remain challenging. The observed limitations appear to be primarily related to anatomical and radiographic complexity rather than to reference annotation inaccuracy or algorithmic instability.

## Introduction

Cephalometric analysis plays a central role in orthodontic diagnosis, treatment planning, and evaluation of craniofacial relationships. By identifying specific anatomical landmarks on lateral cephalometric radiographs, clinicians are able to quantify skeletal, dental, and soft tissue relationships using established analyses such as Steiner, Tweed, and others. Traditionally, this process has been performed manually through tracing techniques, either on acetate sheets or using conventional digital tools. Despite its clinical value, manual cephalometric tracing is time-consuming and highly dependent on operator expertise, making it subject to both inter-observer and intra-observer variability (1 , 2). Landmark identification, in particular, represents the main source of measurement error in cephalometry. With the rapid development of artificial intelligence (AI), automated cephalometric landmark detection has emerged as a promising solution to streamline clinical workflows while maintaining or even improving diagnostic accuracy (3 , 4). Deep learning algorithms trained on large datasets of annotated cephalometric images are now capable of recognizing anatomical patterns and automatically predicting landmark positions (5 , 6). These systems aim to reduce operator-dependent variability, accelerate analysis time, and provide standardized measurements. However, before such tools can be integrated into routine clinical practice, their reliability, precision, and clinical validity must be rigorously evaluated against expert manual annotations considered as the reference standard (7 , 8). Recently, WebCeph has been introduced as an AI-based orthodontic and orthognathic cloud platform capable of automatically performing cephalometric analysis from uploaded radiographs (9). The system uses deep learning models trained on extensive annotated datasets to identify anatomical landmarks and generate cephalometric measurements in a fully automated manner. In addition to cephalometric tracing, the platform offers tools for patient record organization and standardized orthodontic assessment. Despite its growing adoption, the accuracy and reproducibility of WebCeph's automated landmark detection remain insufficiently documented in clinical validation studies, highlighting the need for independent evaluation before routine clinical approval. Therefore, the present study aims to evaluate the accuracy, precision, and stability of cephalometric landmark detection performed by an AI-based system (WebCeph) in comparison with expert manual annotations used as the ground truth reference. By analyzing coordinate-based discrepancies between AI predictions and expert tracings, this study seeks to provide objective evidence regarding the clinical reliability of automated cephalometric analysis. - Study objectives: Primary objective To evaluate the accuracy and precision an of AI-based cephalometric landmark detection software WebCeph relative to expert manual annotations used as ground truth reference. Secondary objectives To characterize the landmark-specific stability of AI localization behavior across radiographs using the coefficient of variation (CV). To evaluate the reliability of the ground truth manual annotations through inter-observer agreement analysis using interclass correlation coefficients and Bland-Altman methods. - Study Design: This study is a quantitative, analytical, observational study based on retrospective or prospective analysis of lateral cephalograms. We evaluated the performance of the AI-based software by comparing it to expert manual annotations used as the gold standard.

## Materials and Methods

This study was conducted at the Centre de Consultations et de Traitements Dentaires (CCTD), Ibn Sina University Hospital in Rabat, Morocco, between June 2025 and January 2026. The study was carried out in accordance with the principles of the declaration of Helsinki (10), and all applicable regulation regarding data protection and patient confidentiality were strictly respected. All radiographic data were anonymized prior to analysis to ensure patient privacy. - Selection of the lateral cephalometric radiographs: The sample comprised lateral cephalograms of 100 patients (mean age: 15.9 ± 5.8 years; age range: 14.0 years; 72 females and 28 males), randomly collected from the CCTD database of the Department of Dentofacial Orthopedics and Orthodontics, Ibn Sina University Hospital in Rabat, Morocco (Table 1).

Table 1

All cephalograms were acquired using a CareStream Dental CS 9600 imaging system (CareStream Dental LLC, Atlanta, GA, USA) using software (CS Imaging 8.0.27) in high resolution (DPI 300) TIFF format with an image dimensions of 2603 × 3018 pixels. To ensure measurement consistency, all images used in the current study were verified to maintain their original resolution and DPI throughout the annotation and analysis process. all images exhibiting signs of rescaling or missing DPI metadata were excluded from the dataset. Radiographs were meticulously selected to be free from any artifacts. Clear anatomical landmarks, patient in natural head position, shadows of the cephalostat and teeth in centric occlusion with Frankfurt plane parallel to floor were the criteria upon which the radiographs were checked for eligibility to be included in the current study. - Measurements' production: All the included cephalograms were assessed with the 2 anatomical landmarks detection methods (digital manual method and AI based technique). The digital manual method, regarded as the gold standard for the study, involved manual placement of landmarks. Whereas with AI-based software, the cephalometric points were localized automatically by the software WebCephTm over the cephalometry. The gold standard (the manual method) use for tracing All images were annotated ("traced") independently by three clinical orthodontists (with four, five and 15 years of experience, respectively), yielding a manual annotation of 20 cephalometric landmark positions as shown in Table 2.

Table 2

All landmarks were chosen to mark common structures used in cephalometric evaluations such as Steiner or Tweed analysis. Digital cephalometric tracing using AutoCAD (version 2025.1.2, Autodesk Inc., USA) software involved several steps. First, the patient's information and the digital cephalogram were uploaded into the software, ensuring proper orientation and scaling. Next, the user identified and manually marked the 20 anatomical landmarks, using the program's tools. To avoid bias in the tracing, the three operators executed, independently, an average of 20 tracings per day with AutoCAD (conventional digital technique) under the same conditions to avoid lack of concentration and fatigue. Inter-annotator agreement was assessed by computing the Euclidean distance between corresponding landmark coordinates, as well as interclass correlation coefficient (ICC) and Bland-Altman analysis. These metrics were used to evaluate the reliability and reproducibility of the manual annotations as ground truth. Coordinates extraction A custom web-based application, " Analyseur de Points Anatomiques -Radiologie Dentaire (APARD) ", was developed by the authors and deployed using the Streamlit framework. The application allows users to upload radiographic images with annotations and automatically extract anatomical landmark coordinates. Coordinates are initially retrieved in pixel units using the conventional image coordinate system, where the origin (0,0) is located at the top-left corner of the image. To ensure consistency with the coordinate convention used by WebCeph, APARD performs an automatic coordinate transformation, translating the origin to the Sella (S) landmark, such that all landmark coordinates are expressed relative to S. Pixel coordinates are subsequently converted to millimeters using a scaling factor obtained from the WebCeph calibration module. In the present dataset, the calibration factor was 4,545 pixels per millimeter, derived from a 10-mm reference ruler. This processing ensures direct numerical comparability between manually extracted coordinates and WebCeph-exported coordinates. To validate the accuracy and reliability of the coordinate extraction pipeline, a verification test was performed using 10 randomly selected radiographs. For each case, landmark coordinates exported directly from WebCeph (Excel format) were independently extracted using APARD from the corresponding annotated images. Identical coordinate values were obtained across all tested landmarks, confirming the correctness of coordinate extraction, coordinate transformation, and pixel-to-millimeter conversion implemented in the application. This automated workflow minimizes manual transcription errors, improves reproducibility of coordinate extraction, and enables standardized and reliable comparison between different annotation sources, thereby strengthening the methodological robustness of the study. Employment of the AI system In the fully automated AI version, the process started with uploading a lateral cephalometric radiograph to the platform. The AI used by Webceph (WEBCEPH, Artificial Intelligence Orthodontic &amp;Orthognathic Cloud Platform, South Korea, 2020) operates through deep learning algorithms that have been trained on large datasets of annotated cephalometric images. These neural networks were designed to recognize anatomical patterns, enabling the AI to detect and trace key cephalometric landmarks. The system used a multi-step process that involved feature extraction, classification, and prediction to locate the landmarks. The AI cross-referenced the radiograph with its vast database, ensuring accuracy by minimizing variability in landmark identification (8). The primary outcome was to compare the accuracy and the precision of the anatomical landmarks' detection by the fully automated software WEBCEPH with the ground truth annotations (conventional digital technique). Sample size calculation The sample size was justified using a precision-based approach for estimating the mean localization error. A total of 100 lateral cephalograms were included. Although 20 landmarks were assessed per image (~2000 landmark observations), these measurements are clustered within radiographs and therefore not statistically independent; consequently, precision for the overall mean error was conservatively based on the number of radiographs (n = 100). Based on pilot variability in localization error (SD 2-3 mm), a sample of 100 radiographs yields an expected 95% confidence-interval half-width of approximately 0.4-0.6 mm for the mean error (depending on the landmark). The inclusion of subjects of different ages and both sexes improves the presentiveness and generalizability of the dataset. Therefore, the selected sample size was considered sufficient to evaluate the accuracy and precision of the automated landmark detection system WebCeph. Statistical methods For each landmark, we defined the localization error as the Euclidean distance between the manually annotated reference coordinates (xm,ym) and the automatically predicted coordinates (xa,ya), expressed in millimeters. Error = [(x_p x_r)² + (y_p y_r)²] Although multiple landmarks were assessed per image, analyses focused on error distributions and summary measures rather than treating individual landmark observations as fully independent samples. Descriptive statistics were computed for each landmark and for the entire dataset, including mean error, standard deviation (SD), median, minimum, maximum, and root square error (RMSE). Accuracy of WebCeph was assessed using the mean and median localization errors, as well as the proportion of landmarks detected within a clinically acceptable threshold of 2 mm (success detection rate). Precision was assessed using the standard deviation and the dispersion of error distributions across images for each landmark, reflecting the stability and consistency of the AI detection behavior. Error distributions were visualized using histograms for global assessment of localization accuracy and boxplots for per-landmark comparison of error dispersion. These visualizations allowed us to evaluate the overall error profile as well as landmark-specific variability across images. Outliers were identified descriptively and analyzed qualitatively to determine whether they were associated with anatomically complex regions rather than random detection failures. In addition to accuracy and precision metrics, we assessed landmark-specific stability of AI performance across images as well and that by using the coefficient of variation (CV) of localization error. Because the AI system produces deterministic outputs, CV was used to quantify the relative dispersion of errors across radiographs for each landmark, rather than test-retest variability. Lower CV values indicate stable, anatomy-driven error patterns, whereas higher CV values reflect increased variability associated with morphologically complex or soft-tissue landmarks. Inter-observer reproducibility of the manual annotations used as ground truth was assessed on a subset of 20 randomly selected cephalograms independently annotated by two expert orthodontists (A.EMD and A.A). For each landmark, we calculated the Euclidean distance between annotations. Agreement was evaluated using the interclass correlation coefficients (ICC) and Bland-Altman analysis was preformed to quantify consistency, systematic bias and limits of agreement. Statistical analyses and graphical visualizations were preformed using Jamovi version 2.6.44 with standard scientific libraries for data analysis and visualization.

## Results

A total of 100 lateral cephalograms were included in the analysis, with 20 anatomical landmarks identified per image, resulting in approximately 2000 landmark measurements. - Inter-observer agreement Inter-observer agreement was excellent. The mean Euclidean distance between manual annotations was 1.53 ± 0.70 mm, indicating low absolute spatial variability between observers. The intraclass correlation coefficient (ICC, two-way random-effects model, absolute agreement) was 0.988, reflecting very high consistency (Table 3).

Table 3

**Figure 1:**
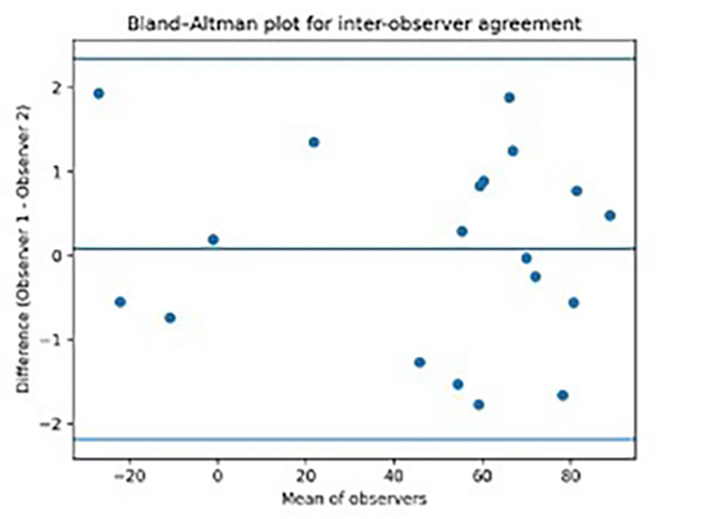
Inter-observer agreement for manual landmark annotation.

Because ICC values may be influenced by between-subject variability and measurement scale, absolute agreement was further assessed using Bland-Altman analysis. This revealed a minimal systematic bias (mean difference: 0.08 mm) and narrow limits of agreement (2.19 to +2.34 mm), confirming high inter-observer agreement without evidence of systematic annotation bias (Fig. 1).

1

- Accuracy and precision The overall mean localization error was 3.16 mm, with a median error of 2.46 mm, and a global root mean square error (RMSE) of 4.11 mm. Considering the clinically accepted cephalometric threshold of 2 mm, only 42.5% of landmark detections fell within the acceptable range, while the remaining detections exceeded this limit. The maximum observed localization error reached 22.88 mm. The distribution of localization errors demonstrated a right-skewed pattern, with the majority of landmark predictions concentrated at lower error values, while a limited number of large errors contributed disproportionately to the elevated mean and maximum values. Per-landmark analysis revealed variability in performance across anatomical structures (Table 4).

Table 4

**Figure 2:**
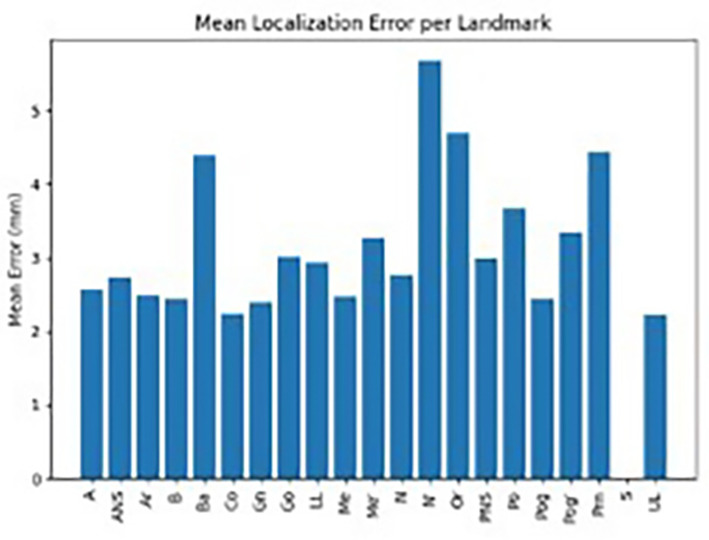
Mean localization error per cephalometric landmark.

**Figure 3:**
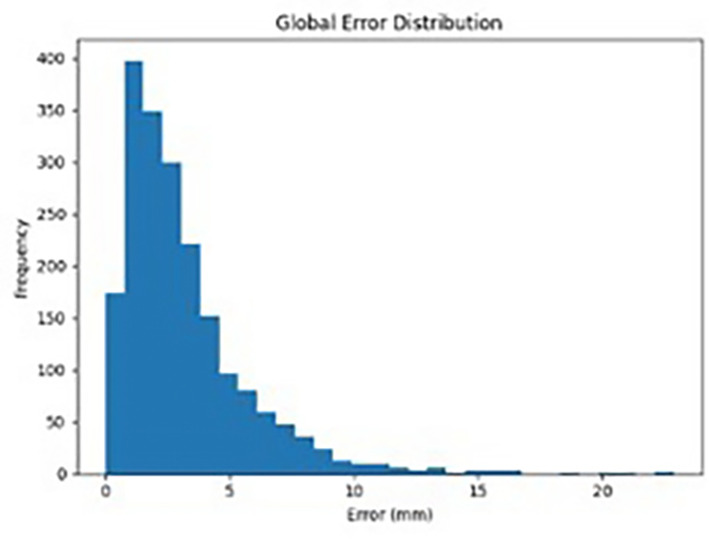
Distribution of Euclidean localization error across landmarks.

Several skeletal landmarks exhibited relatively low mean errors (approximately 2.2-2.5 mm), whereas larger dispersion and higher error values were observed for certain landmarks, particularly those associated with soft tissues or morphologically ambiguous regions. Boxplot visualization highlighted increased variability and the presence of outliers for these landmarks (Figs. 2,3).

2

3

Precision analysis showed moderate dispersion of localization errors, with standard deviation values generally below 3 mm for most skeletal landmarks, indicating relatively consistent localization performance. Finally, a difference in localization error between soft tissue and skeletal landmarks was observed; however, this difference did not reach statistical significance at the 0.05 level (p=0.052) (Table 5).

Table 5

- Landmark-specific stability of AI localization Landmark-specific stability analysis revealed that several skeletal landmarks, such as Po (CV = 0.49) and Point A (CV = 0.69), exhibited low coefficients of variation, indicating stable AI localization behavior across radiographs. In contrast, higher CV values ranging from 0.96 to 1.11 were observed for certain landmarks, particularly those associated with soft tissues or morphologically complex anatomical regions (Table 6).

Table 6

## Discussion

This study evaluates the performance of an artificial intelligence-based system Webceph for automatic cephalometric landmark detection on lateral radiographs by comparing AI-predicted coordinates with expert manual annotations. Prior to assessing AI performance, the reliability of the manual reference standard was verified through an inter-observer agreement experiment, ensuring that the ground truth used for AI evaluation was reproducible and methodologically robust. - Reliability of the ground truth Before interpreting AI performance, we had to verify the consistency of the manual annotations. A subset of 20 radiographs was independently annotated by two experienced orthodontists (A.EMD, A.A), resulting in 399 landmark comparisons. The analysis demonstrated an excellent inter-observer agreement, which indicates low spatial variability between annotators. Furthermore, landmark-wise analysis revealed that agreement was consistent across most anatomical points, although we observed slightly higher dispersion for anatomically complex landmarks such as Basion and Orbital points; a phenomenon that is widely described in the literature (11 - 13) due to superimposition of structures and reduced radiographic contrast. Collectively, these findings confirm that the manual annotations constitute a reliable expert reference while acknowledging the intrinsic uncertainty associated with particular anatomical landmarks. - Global AI performance The performance of WebCeph observed in this study should be interpreted in light of the anatomical nature of cephalometric landmarks rather than solely through global error metrics. Although the overall mean localization error exceeded the 2-mm clinical threshold, this value was disproportionately influenced by only a limited number of anatomically ambiguous points. In contrast, many skeletal landmarks showed errors close to the clinical acceptability range, indicating that the AI system performs reliably when the radiographic contours are well defined, thus its behavior is landmark-dependent. This is consistent with we found in cephalometric AI literature, where performance is usually reported using the 2-mm threshold as a clinical acceptability reference, while acknowledging that this criterion is not always achievable across all landmarks (6 , 14). Even with a rigorously validated reference, the observed errors can remain substantial for specific anatomical points. More importantly, this pattern mirrors what we know from manual cephalometry literature. In fact, several studies show that inter- and intra-observer reliability is generally high for many landmarks, but variability increases for landmarks that are affected by superimposition, or unclear contours, and reduced radiographic contrast, which can limit both human and AI precision in those regions (4 , 12 , 15). Soft-tissue landmarks showed greater error dispersion than skeletal landmarks, although this difference is not statistically significant. This finding is consistent with well-documented challenges in cephalometric analysis. As early as the foundational work of Houston (1983) (12) and Midtgard et al. (1974) (16), skeletal landmarks usually demonstrated superior reproducibility due to the presence of clearly defined bony contours that provide stable reference edges. On the other hand, soft-tissue landmarks depend on profile interpretation, which is inherently more variable, this limitation is further emphasized by Staburn and Danielsen (1982) (17) and Trpkova et al. (1997) (18), who reported increased landmark identification error in regions that are affected by low contrast and overlapping structures. In addition to that, soft-tissue points are also affected by their sensitivity to patient posture, lip tension, and facial expression during image acquisition, these factors introduce more variability even under standardized conditions. These same limitations have been reported in automated cephalometric systems, where studies conducted by Rudolph et al. 1998 (19) and Panesare et al. 2023 (20) have demonstrated lower accuracy and higher dispersion for soft-tissue and profile landmarks compared with skeletal points. Importantly, the persistence of this pattern in the inter-observer validation subset of the present study supports the conclusion that the AI model inherits the same anatomical and radiographic detection challenges observed in human annotation, rather than introducing method-specific errors. Furthermore, the stability analysis demonstrated that landmarks with clear skeletal definition exhibited very stable error patterns across radiographs (e.g., coefficient of variation 0.5), whereas landmarks such as Pog' and Prn showed greater variability (CV 1). This finding indicates that the AI system behaves deterministically, which means its errors are anatomically driven rather than resulting from an algorithmic instability. Similar landmark-specific difficulty patterns are reported in automated cephalometric studies (19 , 20), reinforcing the notion that performance limitations are rooted in image characteristics rather than computational instability. From a clinical perspective, the 2-mm threshold remains a widely used benchmark for cephalometric landmark acceptability, and is commonly employed when reporting success detection rates in deep-learning studies and systematic reviews (6 , 21). However, the literature shows that AI accuracy is landmark-dependent. Consequently, global success metrics may obscure clinically relevant errors affecting specific points. This issue has been emphasized in a recent systematic review by Polizzi and Leonardi 2024 (4), in which they caution that conclusions based purely on a universal 2-mm threshold may overestimate clinical readiness and underscores the need for clinician supervision. Accordingly, clinical usability should be interpreted in relation to the diagnostic task and the specific landmarks required, instead of relying on overall averages alone. This conclusion is concordance with contemporary evaluations (4 , 22) showing heterogeneous performance across landmark categories and recommending cautious, landmark-specific interpretation. Therefore, the present data in our study support a landmark-specific interpretation of clinical readiness: AI performance can be considered more reliable for well-defined skeletal landmarks, while the manual verification remains necessary for morphologically ambiguous or soft-tissue landmarks where both human and AI annotations are more variable. - Role of the custom coordinate-extraction application An important methodological contribution of our study is the development and use of a custom coordinate-extraction application to standardize retrieval and processing of landmark positions. This design choice is justified because cephalometric outcomes are highly sensitive to error sources beyond the AI itself, particularly the technical steps that occur between annotation and analysis. Classic orthodontic methodology work emphasizes that measurement error can arise at multiple stages (identification, recording, and processing) and that controlling these stages is essential for valid comparisons between methods (12). In addition, landmark detection' studies show that variability is landmark-dependent and can be substantial even under controlled conditions, meaning that any extra errors introduced by inconsistent scaling, manual transcription, or non-uniform export formats can distort the "true" error attributed to the AI (18). Evidence from quantitative research workflow by Buscemi et al. 2006 (23) demonstrates that single, manual extraction/handling steps produce more errors than standardized/double-checked extraction processes, which supports the rationale for automating and structuring coordinate handling to reduce avoidable secondary errors. By enforcing uniform unit handling and reproducible export, our application therefore acts as a control layer so that reported discrepancies reflect landmark differences instead of artifacts of data handling. - Limitations and future perspectives The study is limited by the intrinsic variability of certain anatomically complex landmarks and by the dataset size. Expanding the dataset and incorporating multi-expert consensus annotations could further refine ground truth reliability. Future work may also explore incorporating anatomical constraints and multi-scale strategies to specifically address landmarks that present greater ambiguity for both humans and AI systems.

## Conclusions

- WebCeph performance was found to be strongly landmark-dependent. Skeletal landmarks with clear radiographic contours were detected with stable and clinically acceptable accuracy, while soft-tissue and morphologically ambiguous landmarks exhibited greater variability. - The near perfect inter-observer agreement confirms that the ground truth was reliable, and that the observed errors arise from the interaction between the AI model and anatomical complexity rather than from reference noise. These findings suggest that WebCeph can be reliably used for well-defined skeletal landmarks, while cautious interpretation remains necessary for soft-tissue points.

## Figures and Tables

**Table 1:** Demographic and age characteristics of the study sample.

Characteristic	Value
Sample size (N)	100
Sex, n (%)	
Female	72 (72%)
Male	28 (28%)
Age (years)	
Mean ± SD	15.9 ± 5.8
Median	14.0
Minimum	9
Maximum	37
Age group distribution, n (%)	
Young adolescents (≤16 years)	61 (61%)
Adults (>16 years)	39 (39%)

Values are presented as number (percentage) for categorical variables and as mean +- standard deviation (SD), median and range for continuous variables.

**Table 2:** Common Cephalometric Landmarks. a: skeletal landmarks. b: soft-tissue landmarks.

a. Skeletal landmarks.		
Abbreviation	Name	Anatomical definition
S	Sella	Geometric center of the sella turcica
N	Nasion	Most anterior point of the frontonasal suture
Ba	Basion	Most inferior–posterior point on the anterior margin of the foramen magnum
Ar	Articulare	Intersection of the posterior border of the mandibular ramus and the cranial base
ANS	Anterior Nasal Spine	Tip of the anterior nasal spine
PNS	Posterior Nasal Spine	Posterior limit of the hard palate
A	Point A (Subspinale)	Deepest point on the anterior contour of the maxilla
B	Point B (Supramentale)	Deepest point on the anterior contour of the mandible
Pog	Pogonion	Most anterior point of the bony chin
Gn	Gnathion	Most anteroinferior point on the mandibular symphysis
Me	Menton	Lowest point on the mandibular symphysis
Go	Gonion	Most posteroinferior point at the angle of the mandible
Or	Orbitale	Lowest point on the inferior margin of the orbit
Po	Porion	Most superior point of the external auditory meatus
b. Soft-tissue landmarks.		
Abbreviation	Name	Anatomical definition
N′	Soft-tissue Nasion	Most anterior point of the soft tissue overlying the nasion
Prn	Pronasale	Most anterior point of the nasal soft tissue
Sn (Co)	Subnasale	Point at the junction of the columella base and the upper lip
UL	Upper Lip	Most anterior point of the upper lip vermilion
LL	Lower Lip	Most anterior point of the lower lip vermilion
Pog′	Soft-tissue Pogonion	Most anterior point of the soft-tissue chin
Me′	Soft-tissue Menton	Lowest point on the soft-tissue contour of the chin

2

**Table 3:** Inter-observer Reproducibility.

Metric	Value
Mean Euclidean distance (mm)	1.53
SD Euclidean distance (mm)	0.70
ICC (2,1)	0.999
Mean difference (Bland–Altman)	0.079
Upper limit of agreement	2.34
Lower limit of agreement	-2.19

3

**Table 4:** Landmark-wise accuracy and clinical acceptability of AI-based detection.

Landmark	Mean (mm)	SD (mm)	Median (mm)	Max (mm)	SDR ≤ 2 mm (%)
A	2.57	1.78	2.21	8.13	44.0
ANS	2.74	2.09	2.14	11.55	46.0
Ar	2.49	1.68	2.15	7.72	45.0
B	2.44	2.70	1.59	20.18	59.0
Ba	4.39	2.71	3.92	15.69	17.0
Co	2.19	1.73	1.74	10.64	57.0
Gn	2.39	2.29	1.79	16.05	58.0
Go	3.01	2.30	2.65	15.30	36.0
LL	2.93	1.64	2.72	10.82	27.0
Me	2.47	2.27	1.80	16.54	56.0
Me'	3.27	2.84	2.64	21.30	40.0
N	2.77	2.32	2.15	12.20	48.0
N'	5.68	3.52	5.11	18.55	11.0
Or	4.69	3.16	3.56	15.11	17.0
PNS	3.00	2.29	2.58	14.63	36.0
Po	3.67	1.78	3.55	8.96	18.0
Pog	2.44	2.52	1.68	16.14	57.0
Pog'	3.35	3.19	2.23	22.81	43.0
Prn	4.43	3.03	3.89	22.88	20.0
UL	2.22	2.18	1.58	12.02	61.0

4

**Table 5:** Mean and median Euclidean localization errors for skeletal and soft=-tissue cephalomteric landmarks. The difference between groups was evaluated using the Mann-Whitney U test, with statistical significance ser at p < 0,05.

Group	N	Mean error (mm)	Median error (mm)	p-value
Skeletal	1198	2.99	2.34	0.052
Soft tissue	500	3.24	2.65	0.052

5

**Table 6:** Landmark-specific stability of AI localization errors across images (CV analysis).

Landmark	Mean Error (mm)	SD (mm)	CV
A	2.57	1.78	0.69
ANS	2.74	2.09	0.76
Ar	2.49	1.68	0.68
B	2.44	2.70	1.11
Ba	4.39	2.71	0.62
Co	2.19	1.73	0.79
Gn	2.39	2.29	0.96
Go	3.01	2.30	0.76
LL	2.93	1.64	0.56
Me	2.47	2.27	0.92
Me'	3.27	2.84	0.87
N	2.77	2.32	0.84
N'	5.68	3.52	0.62
Or	4.69	3.16	0.67
PNS	3.00	2.29	0.77
Po	3.67	1.78	0.49
Pog	2.44	2.52	1.03
Pog'	3.35	3.19	0.95
Prn	4.43	3.03	0.68
UL	2.22	2.18	0.98

6

## Data Availability

The datasets used and/or analyzed during the current study are available from the corresponding author.

## References

[1] Ibragimov B, Xing L (2017). Fully automated quantitative cephalometry using convolutional neural networks. J Med Imaging (Bellingham).

[2] Kim IH, Kim Y, Han S, Park JW, Kim N (2021). Comparing intra-observer variation and automated cephalometric landmark identification on lateral cephalograms. Sci Rep.

[3] Park JH, Hwang HW, Moon JH, Yu Y, Kim H, Her SB (2019). Automated identification of cephalometric landmarks: Part 1-Comparisons between the latest deep-learning methods YOLOV3 and SSD. Angle Orthod.

[4] Polizzi A, Leonardi R (2024). Automatic cephalometric landmark identification with artificial intelligence: An umbrella review of systematic reviews. J Dent.

[5] Kim YH, Lee C, Ha EG, Choi YJ, Han SS (2021). A fully deep learning model for the automatic identification of cephalometric landmarks. Imaging Sci Dent.

[6] Schwendicke F, Chaurasia A, Arsiwala L, Lee JH, Elhennawy K, Jost-Brinkmann PG (2021). Deep learning for cephalometric landmark detection: systematic review and meta-analysis. Clin Oral Investig.

[7] Hung K, Montalvao C, Tanaka R, Kawai T, Bornstein MM (2020). The use and performance of artificial intelligence applications in dental and maxillofacial radiology: a systematic review. Dentomaxillofac Radiol.

[8] Hwang HW, Moon JH, Kim MG, Donatelli RE, Lee SJ (2021). Evaluation of automated cephalometric analysis based on the latest deep learning method. Angle Orthod.

[9] Yassir YA, Salman AR, Nabbat SA (2021). The accuracy and reliability of WebCeph for cephalometric analysis. J Taibah Univ Med Sci.

[10] (2013). World Medical Association Declaration of Helsinki: ethical principles for medical research involving human subjects. JAMA.

[11] Durão AR, Pittayapat P, Rockenbach MI, Olszewski R, Ng S, Ferreira AP (2013). Validity of 2D lateral cephalometry in orthodontics: a systematic review. Prog Orthod.

[12] Houston WJ (1983). The analysis of errors in orthodontic measurements. Am J Orthod.

[13] McClure SR, Sadowsky PL, Ferreira A, Jacobson A (2005). Reliability of digital versus conventional cephalometric radiology: a comparative evaluation. American Journal of Orthodontics and Dentofacial Orthopedics.

[14] Kim H, Shim E, Park J, Kim YJ, Lee U, Kim Y (2020). Web-based fully automated cephalometric analysis by deep learning. Comput Methods Programs Biomed.

[15] Yang S, Song ES, Lee ES, Kang SR, Yi WJ, Lee SP (2023). Ceph-Net: automatic detection of cephalometric landmarks on scanned lateral cephalograms from children and adolescents using an attention-based stacked regression network. BMC Oral Health.

[16] Midtgård J, Björk G, Linder-Aronson S (1974). Reproducibility of cephalometric landmarks and errors of measurements of cephalometric cranial distances. Angle Orthod.

[17] Stabrun AE, Danielsen K (1982). Precision in cephalometric landmark identification. Eur J Orthod.

[18] Trpkova B, Major P, Prasad N, Nebbe B (1997). Cephalometric landmarks identification and reproducibility: a meta analysis. Am J Orthod Dentofacial Orthop.

[19] Rudolph DJ, Sinclair PM, Coggins JM (1998). Automatic computerized radiographic identification of cephalometric landmarks. Am J Orthod Dentofacial Orthop.

[20] Panesar S, Manoharan G, Alshayji AA (2023). Precision and accuracy assessment of cephalometric analyses performed by deep-learning artificial intelligence. Applied Sciences.

[21] Londoño J, Aboal DG, Peña CG (2023). Accuracy of deep learning models for cephalometric landmark detection: a systematic review and meta-analysis. Dentomaxillofacial Radiology.

[22] Bao P, Lee JS, Kim S (2022). Evaluation of cephalometric landmark detection methods: clinical acceptability thresholds. Journal of Dental Research.

[23] Buscemi N, Hartling L, Vandermeer B, Tjosvold L, Klassen TP (2006). Single data extraction generated more errors than double data extraction in systematic reviews. J Clin Epidemiol.

